# LONGITUDINAL INVESTIGATION OF FACTORS PREDICTING RETIREMENT ADJUSTMENT AMONG RETIREES IN TAIWAN

**DOI:** 10.1093/geroni/igac059.2488

**Published:** 2022-12-20

**Authors:** Shiau-Fang Chao, Wei Ye, Wei-Cheng Liu

**Affiliations:** National Taiwan University, Taipei, Taipei, Taiwan (Republic of China); Nation Taiwan University, Taipei, Taipei, Taiwan (Republic of China); Taiwan Social Welfare League, Taipei, Taipei, Taiwan (Republic of China)

## Abstract

BackgroundRetirement can substantially affect one's lifestyle and self-identity. However, little research has focused on how pre-retirement conditions are associated with retirement adjustment.MethodThis study utilized data from 1989 to 2015 Taiwan Longitudinal Study on Aging. A total of 1,471 cases who experienced retirement between waves and completed the subsequent four year and eight year follow-up surveys after retirement were included for analyses. High life satisfaction and low depressive symptoms represented good retirement adjustment. Multiple regression analysis was applied to test the hypothesized relationships. ResultsThe findings were as follows: 1. Retirees experienced increased depressive symptoms and worsened family relationships when transiting from pre-retirement to post-retirement.2. High cognition functioning, ideal family relationships and sufficient financial resources before retirement were consistently associated with few depressive symptoms and high life satisfaction, both at four and eight year follow-ups. 3. Of the 10 different leisure activities investigated, physically active leisure activities such as walking and participating group exercise before retirement predicted few depressive symptoms and high life satisfaction four years after retirement, but not eight year follow-up. DiscussionCognitive functioning, financial status, and family relationship before retirement can significantly affect both short-term (four years) and long-term (eight years) retirement adjustment, whereas physically active leisure activities only link to short-term retirement adjustment. At policy level, pre-retirement financial security should be addressed and well-prepared. At practitioner level, maintaining ideal cognition level, positive family relationship, and engaging physically active leisure activities should be the central intervention target when serving employees who are approaching retirement.

